# Spatial transcriptomics reveals Inhba/Smad2/E2f4 axis in Lrp2^high^ thecal cell proliferation in androgen-induced PCOS mice

**DOI:** 10.3389/fcell.2025.1633254

**Published:** 2025-08-04

**Authors:** Man Luo, Xiaona Tian, Li Li, Guomei Zhang, Wenzhi Liu, Linlin Mei, Haoran Li, Xiaoyan You, Dongmei Zhang, Mengsi Zhou, Cheng Xiao, Jianping Ye, Xiaofeng Yang

**Affiliations:** 1 Department of Obstetrics and Gynecology, Zhengzhou Central Hospital Affiliated to Zhengzhou University, Zhengzhou, China; 2 Zhengzhou Key Laboratory of Endocrine Metabolism and Immunity in Polycystic Ovary Syndrome, Zhengzhou Central Hospital Affiliated to Zhengzhou University, Zhengzhou, China; 3 Institute of Muscle Biology and Growth, Research Institute for Farm Animal Biology (FBN), Dummerstorf, Germany; 4 Institute of Agricultural and Environmental Sciences, Rostock University, Rostock, Germany; 5 Institute of Trauma and Metabolism, Zhengzhou Central Hospital Affiliated to Zhengzhou University, Zhengzhou, China; 6 Tianjian Laboratory of Advanced Biomedical Sciences, Academy of Medical Sciences, Zhengzhou University, Zhengzhou, China

**Keywords:** polycystic ovary syndrome, spatial transcriptomic, thecal cell, androgen, proliferation

## Abstract

**Background:**

Polycystic ovary syndrome (PCOS) is a common endocrine disorder characterized by elevated androgen levels and impaired follicular development. A hallmark of PCOS is the excessive proliferation of thecal cells (TCs), which are critical for androgen production. However, the molecular mechanisms underlying this aberrant cellular expansion remain incompletely understood.

**Methods:**

A DHEA-induced mouse model was used to recapitulate the hormonal and ovarian features of human PCOS. Spatial transcriptomics was employed to profile gene expression in ovarian tissue at cellular resolution. Differential expression analysis, pathway enrichment, and spatial co-localization were performed to identify regulatory networks. Functional assays were conducted in cultured TCs using siRNA-mediated knockdown of target genes, and cell proliferation and cell cycle progression were evaluated using EdU incorporation and flow cytometry.

**Results:**

Spatial transcriptomic profiling revealed widespread transcriptional changes in the ovaries of PCOS mice, including a marked expansion of a TCs subpopulation with high Lrp2 expression. This subset exhibited enhanced activity in genes involved in androgen synthesis and cell cycle regulation. A signaling axis comprising Inhba, Smad2, and E2f4 was identified as a key regulator of this proliferative response, with all three genes co-expressed in the affected regions. Knockdown of any component of this axis significantly suppressed TCs proliferation *in vitro*, with the greatest effect observed upon Inhba silencing.

**Conclusion:**

The Inhba/Smad2/E2f4 axis contributes to thecal cell hyperplasia and androgen excess in PCOS, and may serve as a mechanistic entry point for further investigation into the regulation of TCs proliferation in this disorder.

## Introduction

Polycystic ovary syndrome (PCOS) is a complex endocrine disorder affecting approximately 10%–15% of women of reproductive age worldwide and represents the leading cause of infertility and hyperandrogenism (Teede et al., 2018). It is characterized by ovarian dysfunction, hyperandrogenism, insulin resistance and the presence of multiple small follicles that fail to mature (Lizneva et al., 2016). Despite its high prevalence, the molecular mechanisms underlying PCOS remain incompletely understood, with both genetic and environmental factors contributing to its clinical heterogeneity (Goodarzi et al., 2011; Rudnicka et al., 2021; Rosenfield and Ehrmann, 2016; Aboeldalyl et al., 2021). Elucidating the pathways that drive ovarian dysfunction and excessive androgen production is essential for the development of more effective therapeutic strategies.

A defining feature of PCOS ovaries is the hyperplasia of thecal cells (TCs), which are primarily responsible for androgen production within follicles (Rosenfield and Ehrmann, 2016; Richards et al., 2018; Vlieghe et al., 2023). Increased proliferation and activity of TCs contribute to elevated androgen levels, leading to impaired folliculogenesis and anovulation (Fiorentino et al., 2023). Although several pathways involved in TCs steroidogenesis have been identified (Li et al., 2021), the molecular mechanisms underlying TCs hyperproliferation remain largely unclear. Recent advances in transcriptomic technologies, particularly spatial transcriptomics (ST), now enable the investigation of tissue-specific gene expression and offer new opportunities to uncover regulatory mechanisms associated with PCOS pathophysiology (Asp et al., 2020; Vandereyken et al., 2023).

In PCOS research, dehydroepiandrosterone (DHEA)-induced animal models are widely employed to replicate the hormonal disturbances and ovarian features observed in human patients (Stener-Victorin et al., 2020; Corrie et al., 2021). These models exhibit hallmark characteristics of the disorder, including hyperandrogenism, anovulation, and the presence of polycystic ovaries, closely resembling the human condition (Luo et al., 2021; Luo et al., 2020). Spatial transcriptomic profiling in such models enables the detection of region-specific gene expression patterns within ovarian tissue, providing insight into the local molecular landscape that contributes to PCOS pathogenesis (Rao et al., 2021).

Spatial transcriptomics was employed to characterize gene expression alterations in the ovaries of DHEA-induced PCOS mice. Significant transcriptional changes were observed across distinct ovarian cell clusters, with the most notable alterations detected in TCs population. A previously uncharacterized subpopulation of Lrp2^high^TC was identified, which was markedly expanded and exhibited elevated proliferative activity in PCOS ovaries. Further mechanistic investigation revealed that the proliferation of Lrp2^high^TC was regulated by the INHBA/Smad2/E2f4 signaling axis, implicating this pathway as a key contributor to the pathophysiology of PCOS.

Inhibin Subunit Beta A (INHBA) encodes a protein involved in the regulation of folliculogenesis and steroidogenesis through the transforming growth factor-beta (TGF-β) signaling pathway (Hedger et al., 2011). SMAD family member 2 (Smad2) is a key mediator of TGF-β signaling, which controls cellular processes such as proliferation, differentiation, and apoptosis (Massagué, 2012). E2F transcription factor 4 (E2f4) is a cell cycle regulator that promotes S-phase entry (Wang Y. et al., 2022). Although each of these genes has been implicated in reproductive function, their coordinated role in thecal cell proliferation within the context of PCOS has not been previously described. In this study, we demonstrate that the concerted activity of Inhba, Smad2, and E2f4 is essential for the aberrant proliferation of thecal cells in the DHEA-induced PCOS model.

We hypothesized that the Inhba/Smad2/E2f4 axis functions as a key molecular driver of TCs proliferation, contributing to the expansion of the Lrp2^high^ subpopulation. To test this, we performed functional assays using siRNA-mediated knockdown to evaluate the role of this signaling axis in regulating TCs proliferation. The results consistently support the involvement of this pathway in the molecular mechanisms underlying TCs hyperplasia in PCOS.

## Materials and methods

### Animal model and induction of PCOS

25-day-old female ICR mice were purchased from Liaoning Changsheng Biotechnology Co., Ltd and housed under controlled conditions (12-hour light/dark cycle, temperature 22°C ± 2°C) with free access to food and water. DHEA was dissolved in 10% sesame oil (in 95% ethanol) and administered daily to each mouse in the model group at 6 mg/100 g body weight in 0.1 mL for 20 days. Control mice were injected with vehicle in the same way. Ovaries from mice in the model group were collected at the end of the DHEA administration period. For the control group, ovaries were obtained from mice at the same estrous stage as those in the treatment group to ensure physiological comparability. All samples were immediately frozen in OCT compound for ST.

### Hormone measurements

Testosterone levels were quantified in both mouse serum and ovarian tissue lysates to evaluate the androgenic status of the DHEA-induced PCOS model. Serum was collected via orbital bleeding, and ovarian tissues were homogenized in PBS followed by centrifugation to obtain supernatants. Testosterone concentrations were measured using commercial ELISA kits (Cusabio. China), following the manufacturer’s instructions.

### Spatial transcriptomics

As describe previously, the 10 × Genomics Spatial Transcriptomics platform was used to map gene expression across entire tissue sections, revealing cellular spatial organization and function (Luo et al., 2024). Ovaries from three biological replicates per group (n = 3) were collected, embedded in OCT, sectioned at 10 μm thickness using a Leica CM1950 cryostat, and mounted onto Visium spatial gene expression slides according to the manufacturer’s protocol.

Following tissue permeabilization, mRNA transcripts were captured by spatially barcoded oligonucleotides on the slide surface. Reverse transcription was then performed to synthesize cDNA incorporating spatial barcodes, enabling localization of gene expression to specific tissue regions. Sequencing libraries were generated and subjected to high-throughput sequencing on the Illumina platform.

Raw data processing, including alignment, barcode assignment and gene quantification was performed using Space Ranger (10x Genomics). Quality control filtering was applied to exclude spots with >10% mitochondrial gene content or fewer than 100 detected genes. The number of retained and excluded spots for each sample is summarized in Supplementary Table S1. Unsupervised clustering was conducted in Seurat (v4.0) to identify distinct transcriptional clusters. UMAP was used for dimensionality reduction and visualization of spatially distinct cell populations across control and PCOS ovaries. Key quality control metrics, including total spots under tissue, median reads per spot, and sequencing saturation, are summarized in Supplementary Table S2.

### Differential gene expression and pathway enrichment analysis

Differential gene expression analysis between control and PCOS-like ovary was conducted using Seurat’s FindMarkers function. Genes with an adjusted p-value <0.05 and log2 fold change (FC) > 1 were considered significantly differentially expressed. Gene Ontology (GO) and Kyoto Encyclopedia of Genes and Genomes (KEGG) pathway enrichment analyses were performed using the clusterProfiler package (v4.0) in R. Gene Set Enrichment Analysis (GSEA) was conducted using the fgsea package to identify significantly enriched biological processes and signaling pathways.

#### Gene sets scoring

The Area Under the Curve (AUC) scores for each predefined gene set were calculated using the AUCell package in R, a tool specifically designed to evaluate the relative expression of gene sets at the single-cell level. AUCell assesses whether each cell within a cluster expresses a particular gene set above a given threshold, providing an AUC score that reflects the activation level of that gene set within individual cells.

#### Co-localization analysis

Spatial co-localization of Inhba, Smad2 and E2f4 was analyzed using Visium spatial transcriptomic data. Co-localization spots were identified based on the simultaneous expression of all three genes within the same spatial coordinates. Co-localization quantification was conducted using the Seurat, tidyverse, and ggplot2 packages in R.

#### Cell culture and treatment

Primary mouse TCs (Procell, Cat. No.CP-M205) were cultured in DMEM/F12 medium supplemented with 10% fetal bovine serum and 1% penicillin/streptomycin at 37°C in a humidified 5% CO_2_ atmosphere. For DHEA treatment, TCs were exposed to 100 μM DHEA dissolved in DMSO for 12 h, while control cultures received an equivalent volume of DMSO without DHEA.

#### siRNA transfection

For functional assays, thecal cells were transfected with small interfering RNAs (siRNAs) targeting Inhba (Santa Cruz, Cat. No. sc-39783), Smad2 (Santa Cruz, Cat. No. sc-44338), or E2f4 (Santa Cruz, Catalog No. sc-35248) using Lipofectamine 3,000 (Invitrogen, Cat. No. L3000015) according to the manufacturer’s instructions. A non-targeting siRNA (Santa Cruz, Cat. No. sc-36869) was used as a negative control.

#### Immunofluorescence

Cells were fixed in 4% paraformaldehyde, permeabilized with 0.2% Triton X-100, and blocked with 5% BSA. Primary antibodies against Inhba (1:200; Santa Cruz, Cat. No. sc-166503), Smad2 (1:300; Santa Cruz, Cat. No. sc-7960), and E2f4 (1:300; Santa Cruz, Cat. No. sc-69685) were incubated overnight at 4°C. After washing, cells were incubated for 1 h at room temperature with species-specific secondary antibodies conjugated to fluorescent dyes ((Thermo Fisher, United States, and Abcam, United Kingdom). The following fluorophore-conjugated antibodies were used: Alexa Fluor 488-conjugated goat anti-rabbit IgG (Cat. No. A-11034; green) for Inhba, Alexa Fluor 555-conjugated goat anti-rabbit IgG (Cat. No. ab150078; yellow) for Smad2, and Alexa Fluor 647-conjugated goat anti-rabbit IgG (Cat. No. A-21246; red) for E2f4. Nuclei were counterstained with DAPI (Thermo Fisher, Cat. No. D1306). All secondary antibodies were highly cross-adsorbed to minimize nonspecific binding. Images were acquired using a confocal microscope (Zeiss, Germany).

### EdU incorporation assay

TCs proliferation was assessed using the EdU Cell Proliferation Kit (Beyotime, China). TCs were incubated with 10 μM EdU for 2 h, followed by fixation and detection of EdU incorporation according to the manufacturer’s instructions. Images were captured using a fluorescence microscope (Nikon, Japan), and the percentage of EdU-positive cells was quantified using ImageJ software.

### Flow cytometry for cell cycle analysis

Cell cycle progression in thecal cells was assessed by flow cytometry using a cell cycle and apoptosis detection kit (Beyotime, China), following the manufacturer’s instructions. Briefly, adherent cells were harvested by trypsinization, centrifuged, and washed with pre-chilled PBS. Cells were fixed in 70% ethanol at −20°C for 2 h, rinsed with PBS, and stained with 50 μg/mL propidium iodide and 100 μg/mL RNase A. The distribution of cells across G1, S, and G2/M phases was analyzed on a BD Biosciences flow cytometer using FlowJo software. Three independent biological replicates (n = 3) were included for each experimental group. Each replicate was processed and analyzed separately, without averaging across technical replicates.

### Quantitative real-time PCR

Total RNAs from ovaries or TCs were applied to synthesize cDNA and then amplified to determine the expression levels of Inhba, Betaine-homocysteine S-methyltransferase (Bhmt), Hydroxysteroid (17-beta) dehydrogenase 7 (Hsd17b7), Inhibin subunit beta B (Inhbb), 7-dehydrocholesterol reductase (Dhcr7), Smad2 and E2f4. Relative gene expression was calculated using the 2^−ΔΔCt^ method, normalized to Gapdh as the internal control. The primer sequences were listed in Table 1.

**TABLE 1:** The primer sequences of qRT-PCR.

Gene	Forward primer	Reverse primer	Amplicon size (bp)
Inhba	TCCTGCTGCCTTCACCATCT	ACTGGTCCTGGGTGGGTTAT	120
Bhmt	TGGTGGATGAGGACGAGATG	AGGATGTCCTGGGCTTGTTT	115
Hsd17b7	GCTGGTGGTTTTCGTGTTGG	TCCATCCATCAGCCAACTCC	125
Inhbb	CAGGCTTCCCTGGAACTGTA	TGGACAGCTCAGATGTTGCC	110
Dhcr7	CCTGGACACTACCGACTCTG	GGGCACTCTGGAAGTTCTTG	115
Smad2	GAGTGACCGCCTCAGACACTT	TGGCAACATAGCGGATGACTC	130
E2f4	CGGCACTTGTTTCTGACGTT	AGTGCTGCTTCCATTTGTCG	125
Gapdh	TGTGTCCGTCGTGGATCTGA	TTGCTGTTGAAGTCGCAGGAG	140

### Western blotting

After extraction and quantification, proteins from TCs were subjected to SDS-PAGE and subsequently transferred onto PVDF membranes. The membranes were then incubated overnight with primary antibodies against Inhba (1:1,000, Proteintech, United States), Smad2 (1:2,000, Proteintech, United States), E2f4 (1:2,000, Proteintech, United States), or Gapdh (1:5,000, Proteintech, United States), followed by treatment with an HRP-conjugated secondary antibody. Protein signals were visualized using an enhanced chemiluminescence substrate.

### Statistical analysis

All data are presented as mean ± SD. Statistical analyses were performed using GraphPad Prism (v9.0). Differences between groups were analyzed using unpaired Student’s t-tests or one-way ANOVA followed by Tukey’s post-hoc test, as appropriate. pvalue <0.05 were considered statistically significant.

## Results

### Spatial transcriptomic profiling of ovarian tissue reveals cell cluster alteration in DHEA-induced PCOS model

**FIGURE 1:**
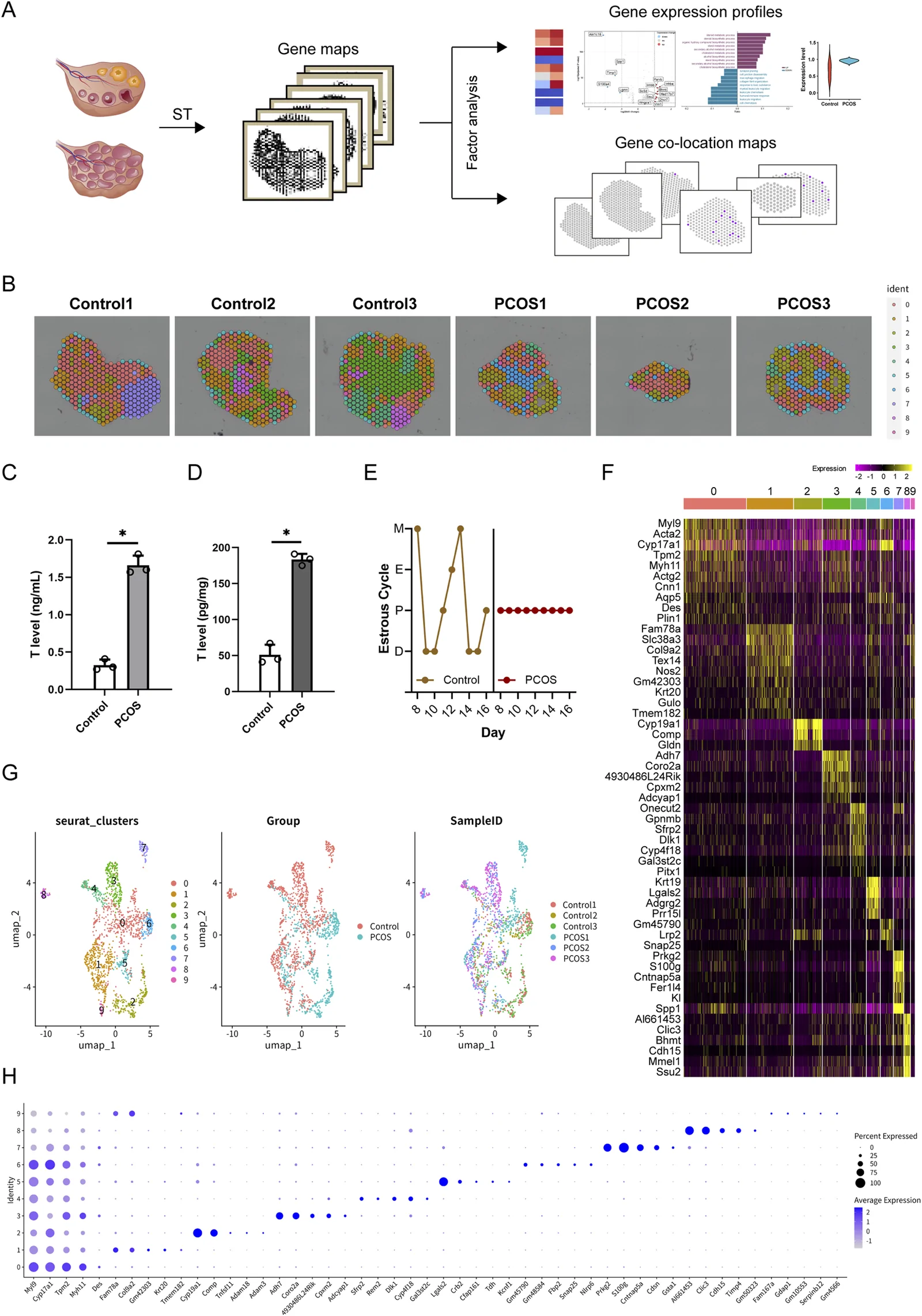
Spatial transcriptomic analysis of ovarian tissue from control and DHEA-induced PCOS mice. **(A)** Schematic representation of spatial transcriptomic analysis workflow for ovarian tissues. **(B)** Spatial mapping of gene expression clusters in control and PCOS ovaries. **(C)** Serum testosterone levels in control and PCOS mice. **(D)** Ovarian tissue testosterone levels. **(E)** Estrous cycle monitoring over 16 consecutive days. PCOS mice exhibited prolonged proestrus. D, diestrus; P, proestrus; E, estrus; M, metestrus. **(F)** Heatmap showing the top 10 marker genes for each cluster across control and PCOS samples. **(G)** UMAP plots illustrating cluster distribution for seurat_clusters, group and SampleID. **(H)** Dot plot indicating key marker gene expression levels across clusters. Data are represented as mean ± SD, *P < 0.05 by t-test.

Spatial transcriptomics was employed to map cell type alterations in ovarian tissues from the PCOS mice (Figure 1A). Unsupervised clustering identified nine distinct transcriptional clusters across (Figure 1B). Both serum and ovarian testosterone levels were significantly elevated in the PCOS group, confirming a hyperandrogenic state. Moreover, estrous cycle monitoring showed that PCOS mice remained in the proestrus phase, indicating disrupted ovulatory function (Figures 1C–E). In the PCOS samples, the clusters 2, 4, and 6 were markedly expanded, while the clusters 1, 3, and 8 were reduced in sizes, indicating alterations in cell composition within the ovarian microenvironment. Heatmap analysis of the top 10 marker genes for each cluster revealed distinct expression patterns, highlighting potential functional heterogeneity (Figure 1F). UMAP visualization further confirmed this possibility, with PCOS samples enriched in the clusters 2, 4, and 6, and reduced in the clusters 1, 3, and 8 (Figure 1G). The dot plot of cluster markers reinforced the distinct gene expression signatures across clusters (Figure 1H). These results highlight substantial molecular and cellular alterations in ovarian tissue in response to DHEA-induced PCOS, providing insights into the reorganization of cell populations in the disorder.

### Steroid biosynthesis upregulated in PCOS ovary

**FIGURE 2:**
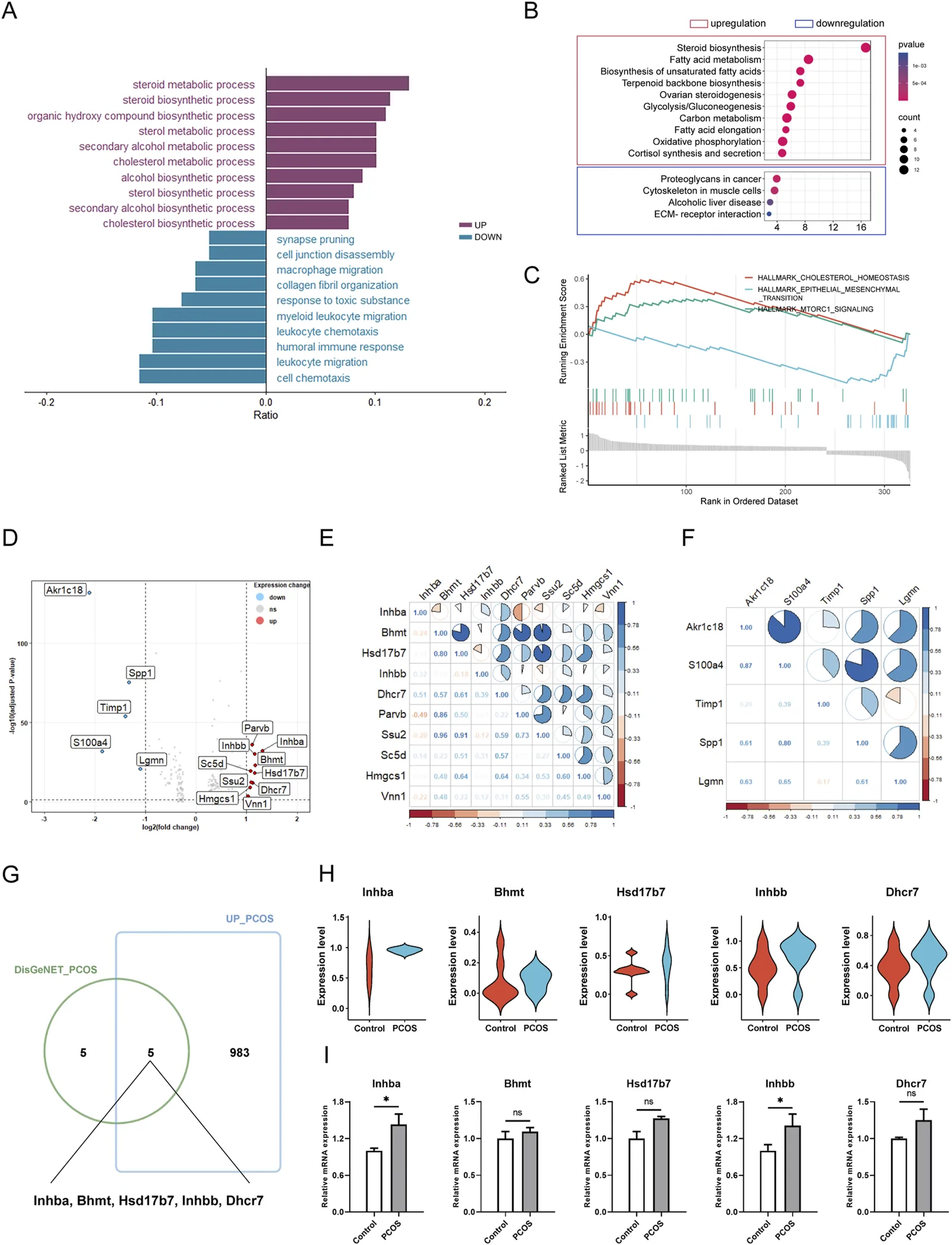
Differential gene expression and functional enrichment analysis in PCOS ovarian tissue. **(A)** GO enrichment analysis of upregulated and downregulated genes in PCOS ovaries. **(B)** KEGG pathway enrichment for differentially expressed genes in PCOS ovaries. **(C)** GSEA showing the top enriched gene sets in PCOS samples. **(D)** Volcano plot of differentially expressed genes between PCOS and control ovaries, highlighting genes with |log2FC| > 1. **(E,F)** Correlation matrices for upregulated and downregulated genes with log2FC > 1 in PCOS ovaries. **(G)** Venn diagram showing the overlap of top upregulated genes with known PCOS-related genes, identifying five common genes. **(H)** Spatial transcriptomic analysis of these five overlapping genes. **(I)** qRT-PCR analysis of the expression of these five genes. Data are represented as mean ± SD, *P < 0.05 by t-test.

To investigate the molecular mechanisms driving cluster alterations, we conducted differential gene expression and functional enrichment analysis between the control and PCOS samples. GO enrichment analysis revealed that genes involved in steroid biosynthesis, steroid metabolism, and cholesterol biosynthesis were significantly upregulated in the PCOS group (Figure 2A). Conversely, downregulated genes were primarily enriched in pathways related to immune responses, including leukocyte migration and chemotaxis. KEGG pathway analysis further identified significant upregulation of pathways involved in steroid biosynthesis and various metabolic processes in PCOS samples (Figure 2B). GSEA analysis supported these findings, revealing enrichment of cholesterol homeostasis and epithelial-to-mesenchymal transition pathways in the PCOS group (Figure 2C). Differential expression analysis between PCOS and control ovaries highlighted several key upregulated genes, including Inhba, Bhmt, Hsd17b7, Inhbb and Dhcr7 (Figure 2D). Co-expression network analysis of these upregulated and downregulated genes revealed strong associations, particularly among genes involved in steroidogenesis (Figures 2E,F). Cross-referencing with the DisGeNET PCOS database identified five overlapping genes Inhba, Bhmt, Hsd17b7, Inhbb and Dhcr7, which were notably elevated in the PCOS group and confirmed by both ST and qRT-PCR validation (Figures 2G–I). Among these, Inhba was the most significantly upregulated gene, suggesting a pivotal role in the pathogenesis of DHEA-induced PCOS.

### Lrp2^high^ TC expansion and localization in primary follicles of PCOS ovaries

**FIGURE 3:**
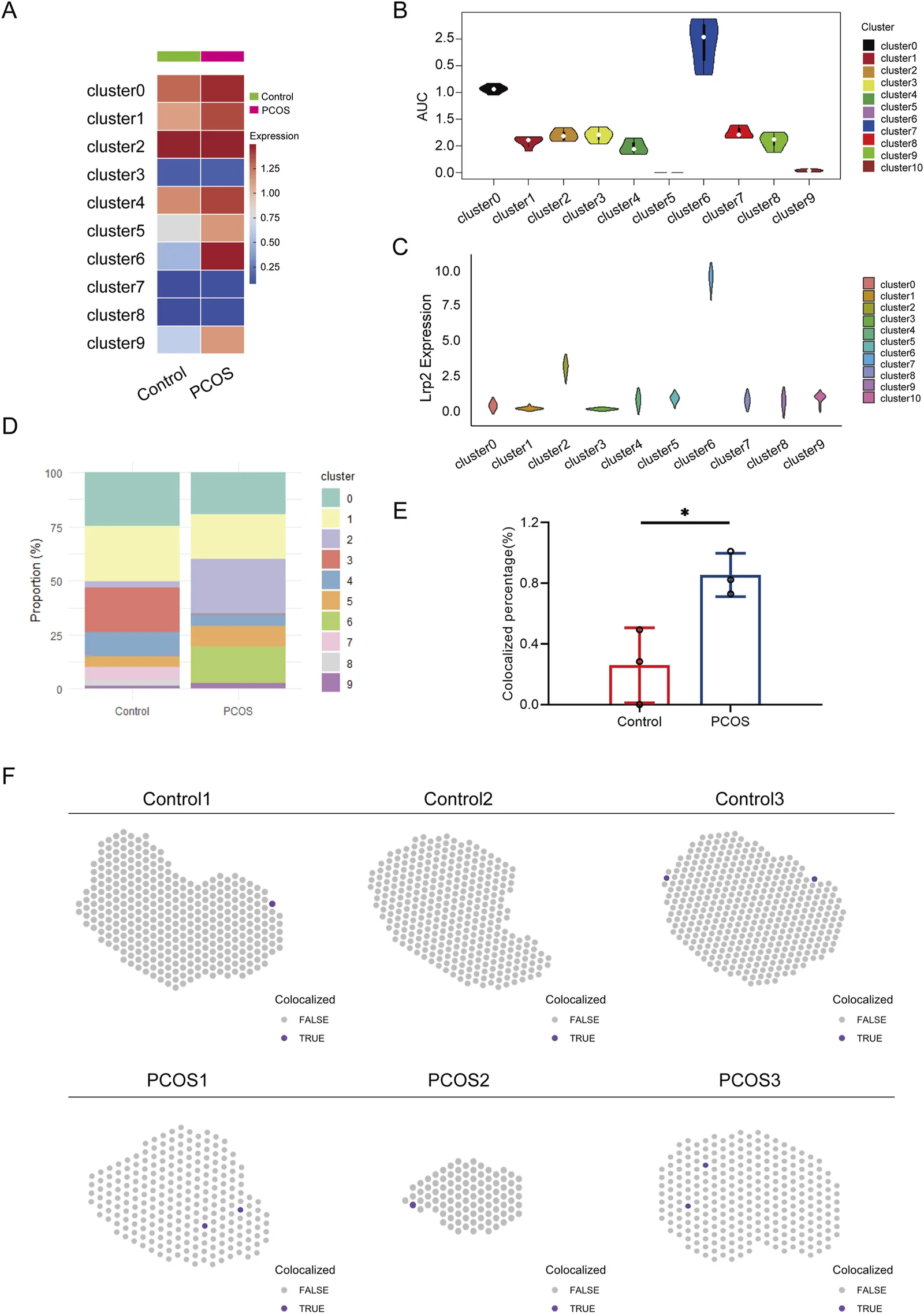
Lrp2^high^TC expansion and localization in primary follicles of PCOS ovaries. **(A)** Heatmap displaying Inhba expression across clusters, with cluster 6 showing increased levels in PCOS ovaries. **(B)** AUC scores confirm cluster 6 as enriched with TCs markers. **(C)** Violin plot illustrating elevated Lrp2 expression in cluster 6 within PCOS ovaries. **(D)** Proportion of cells in cluster 6 is significantly higher in PCOS ovaries. **(E)** Quantification of co-localized regions for Gdf9, Bmp15 and Lrp2, showing a significant increase in PCOS ovaries. **(F)** Spatial co-localization of Gdf9, Bmp15 and Lrp2 in control and PCOS ovaries. Co-expressed spots are marked as “TRUE”.

In PCOS ovaries, cluster 6 was identified as a TCs population through AUC scoring with a TC-specific gene set (Morris et al., 2022) (Figures 3A,B). This cluster, defined as Lrp2^high^ TC, displayed significantly elevated expression of Lrp2 compared to other clusters. Notably, Lrp2^high^ TC was significantly expanded in PCOS samples relative to controls, as demonstrated by cluster composition analysis (Figures 3C,D). Gdf9 and Bmp15 are considered as markers of primary follicles, with their expression being minimal in primordial follicles but undergoing significant upregulation during the primary follicle stage. At this stage, these markers play pivotal roles in granulosa cell proliferation, differentiation, and oocyte-granulosa cell communication, emphasizing their importance in follicle activation and development (Sanfins et al., 2018). The co-localization of Gdf9, Bmp15 and Lrp2 in PCOS samples, shown through quantification and spatial visualization (Figures 3E,F), indicated that the proliferation of Lrp2^high^ TC is concentrated predominantly within primary follicles.

### Proliferative and androgenic features of Lrp2^high^ TC in PCOS ovaries

**FIGURE 4:**
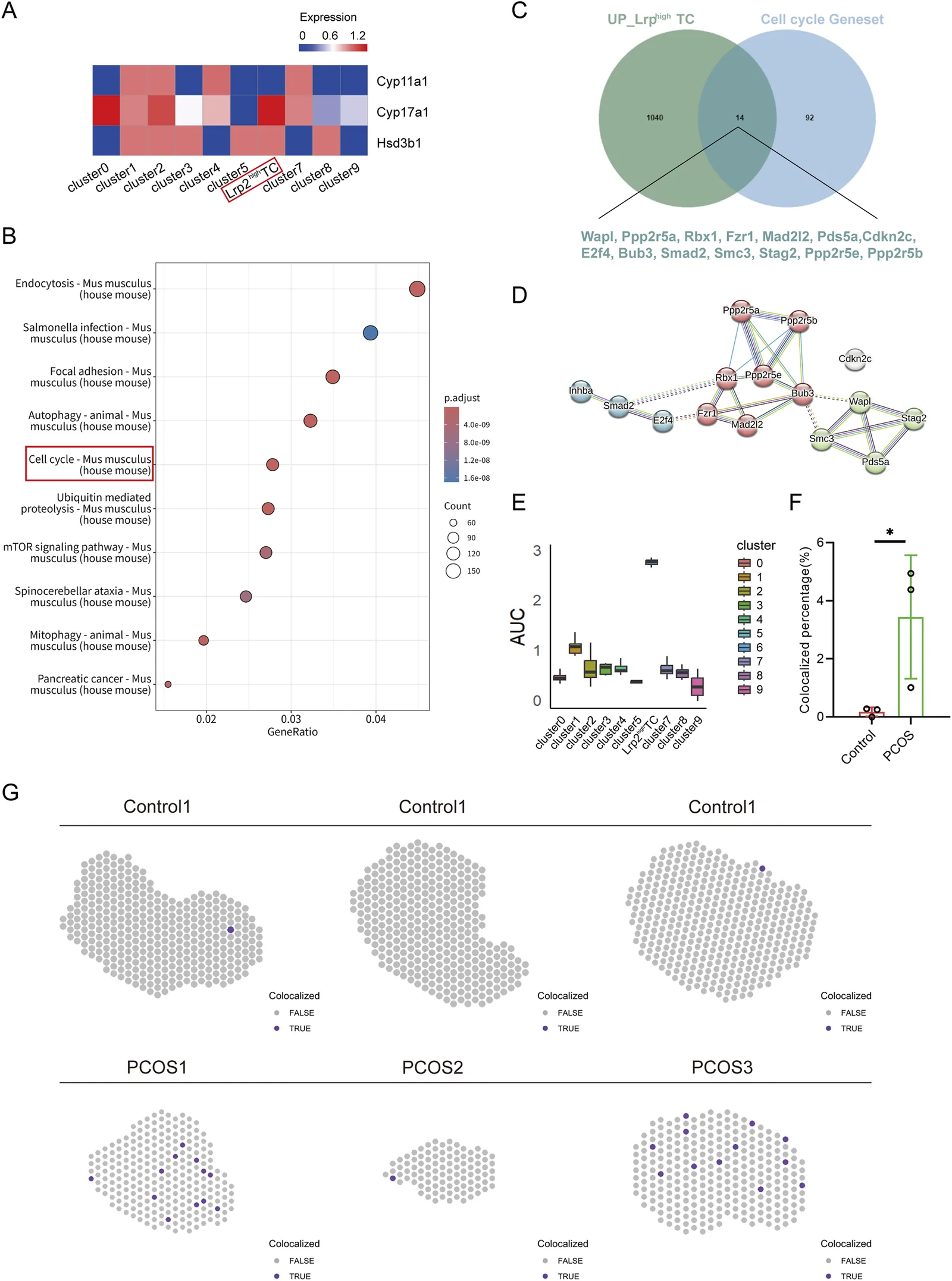
Proliferative and androgenic features of Lrp2^high^TC in PCOS ovaries. **(A)** Expression analysis of androgen synthesis genes Cyp11a1, Cyp17a1, and Hsd3b1 across clusters. **(B)** KEGG pathway enrichment analysis for upregulated genes in Lrp2^high^TC, emphasizing cell cycle regulation. **(C,D)** Intersection and STRING network analysis predict an Inhba/Smad2/E2f4 signaling axis involved in cell cycle regulation within Lrp2^high^TC. **(E)** AUC analysis reveals Lrp2^high^ TC as having the highest co-expression of Inhba, Smad2, and E2f4. **(F)** Quantification of co-localized regions for Inhba, Smad2, E2f4 and Lrp2, showing a significant increase in PCOS ovaries. **(G)** Spatial co-localization of Inhba, Smad2, E2f4 and Lrp2 in control and PCOS ovaries. Co-expressed spots are marked as “TRUE.” Data are represented as mean ± SD, *P < 0.05 by t-test.

The Lrp2^high^ TC population demonstrated enriched expression of key androgen biosynthesis genes, including Cyp17a1 and Hsd3b1, with Cyp17a1 being predominantly expressed (Figure 4A). This suggests that Lrp2^high^ TC are significant contributors to androgen production in PCOS ovaries. KEGG pathway analysis further revealed a notable enrichment of cell cycle-related pathways in Lrp2^high^ TC, indicating a high proliferative potential of this cluster (Figure 4B). Intersection analysis identified 14 core cell cycle-related genes, and STRING network analysis highlighted the Inhba/Smad2/E2f4 signaling axis as the primary driver of Lrp2^high^ TC proliferation (Figures 4C,D). AUC analysis confirmed that Lrp2^high^ TC exhibit the highest co-expression levels of Inhba, Smad2, and E2f4, emphasizing the axis’s role in promoting cell cycle progression within this cluster (Figure 4E). Spatial transcriptomics further corroborated the interactions among Inhba, Smad2, and E2f4 in Lrp2^high^ TC through co-localization analysis. Quantitative analysis of co-localized spots revealed a significantly expanded region of co-localization in PCOS ovaries compared to controls (Figures 4F,G), highlighting the spatial and functional specificity of this proliferative, androgenic cell population.

### Inhba/Smad2/E2f4 signaling drives TCs proliferation

**FIGURE 5:**
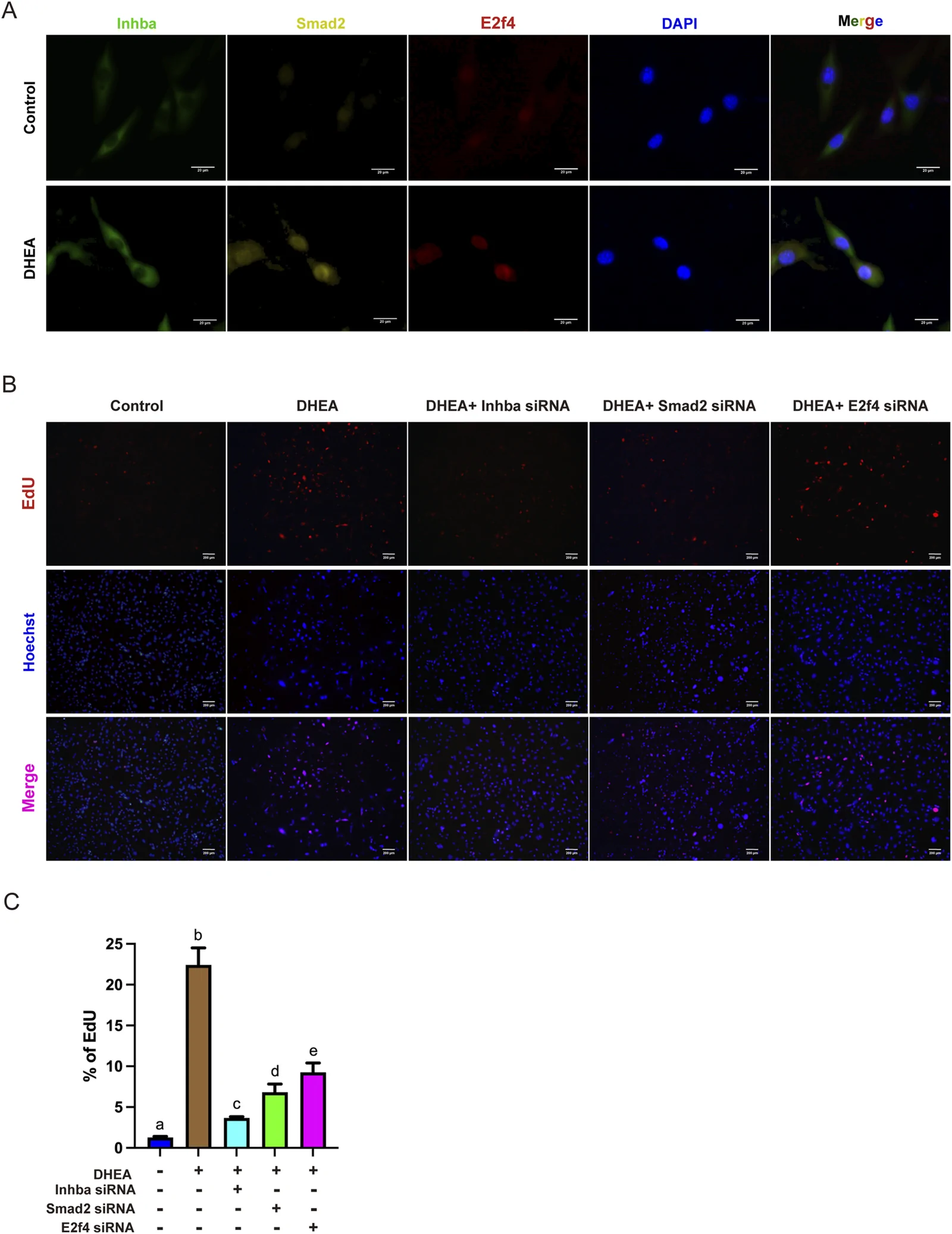
Inhba/Smad2/E2f4 Signaling Promotes Thecal Cell Proliferation. **(A)** Immunofluorescence staining showing the subcellular localization of Inhba (cytoplasmic), Smad2, and E2f4 (nuclear) in primary thecal cells under control and DHEA-treated conditions. DAPI (blue) marks nuclei. Bar = 20 µm. **(B)** Representative EdU staining images of proliferating thecal cells following DHEA treatment and siRNA-mediated knockdown of Inhba, Smad2, or E2f4. Bar = 200 µm. **(C)** Quantification of EdU-positive nuclei across treatment groups. Data are presented as mean ± SD. Different lowercase letters denote statistically significant differences (one-way ANOVA followed by Tukey’s *post hoc* test, P < 0.05).

To investigate the spatial dynamics of the Inhba/Smad2/E2f4 signaling axis in thecal cells under androgenic stimulation, immunofluorescence staining was conducted to assess subcellular localization. As shown in Figure 5A, Inhba was predominantly localized in the cytoplasm, whereas Smad2 and E2f4 were mainly detected in the nucleus, reflecting their canonical roles in intracellular signal relay and transcriptional regulation. Following DHEA treatment, expression levels of all three proteins were notably increased, with enhanced cytoplasmic Inhba signal and corresponding nuclear accumulation of Smad2 and E2f4. This spatial pattern supports the activation of a cytoplasm-to-nucleus signaling cascade in response to androgen stimulation.

To determine the functional relevance of this axis, EdU incorporation assays were performed after siRNA-mediated knockdown of Inhba, Smad2, or E2f4. As shown in Figures 5B,C, DHEA significantly increased the proportion of EdU-positive nuclei, indicating augmented DNA synthesis and proliferation. This proliferative effect was substantially attenuated upon silencing any of the three genes, with Inhba knockdown producing the most pronounced suppression. These findings underscore the critical role of the Inhba/Smad2/E2f4 axis in mediating androgen-driven thecal cell proliferation.

### Inhba/Smad2/E2f4 axis promotes cell cycle progression in TCs

**FIGURE 6:**
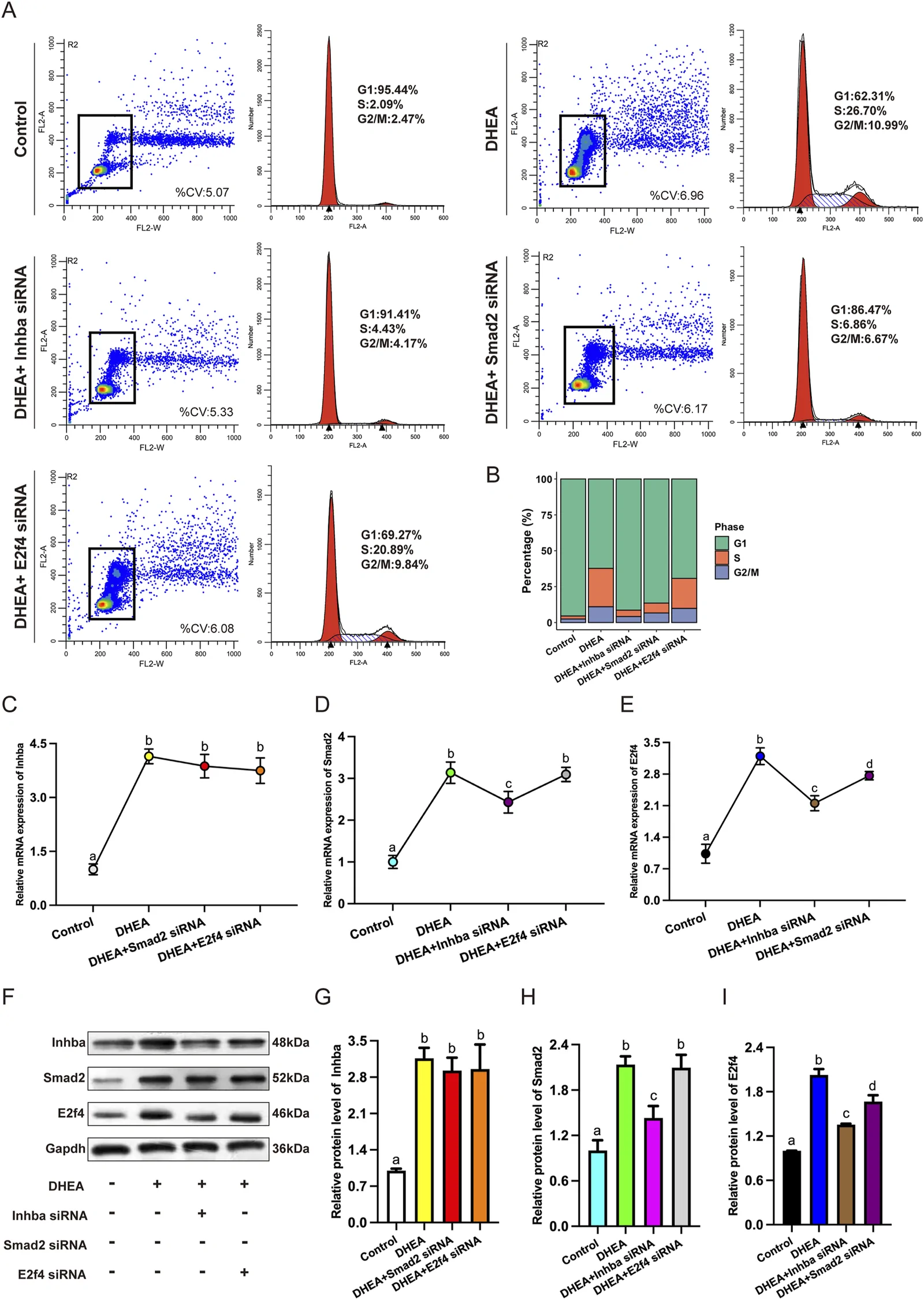
Inhba/Smad2/E2f4 Axis Promotes Cell Cycle Progression in TCs. **(A)** Flow cytometry analysis of cell cycle phases. **(B)** Distribution of cells in G1, S, and G2/M phases (%). **(C–E)** qRT-PCR analysis of Inhba, Smad2, and E2f4 mRNA levels. **(F)** Western blot analysis of protein expression. **(G–I)** Quantification of protein levels for Inhba, Smad2, and E2f4. Data are represented as mean ± SD. Different lowercase letters at the top of each bar denote significant differences among groups (one-way ANOVA followed by Tukey’s *post hoc* test, P < 0.05).

Flow cytometry was next employed to assess cell cycle changes (Figures 6A,B). DHEA treatment significantly decreased the proportion of cells in G1 phase while increasing S and G2/M phases, consistent with cell cycle progression and active proliferation. These effects were reversed upon knockdown of Inhba, Smad2, or E2f4, confirming the requirement of this axis for androgen-driven cell cycle advancement.

**FIGURE 7:**
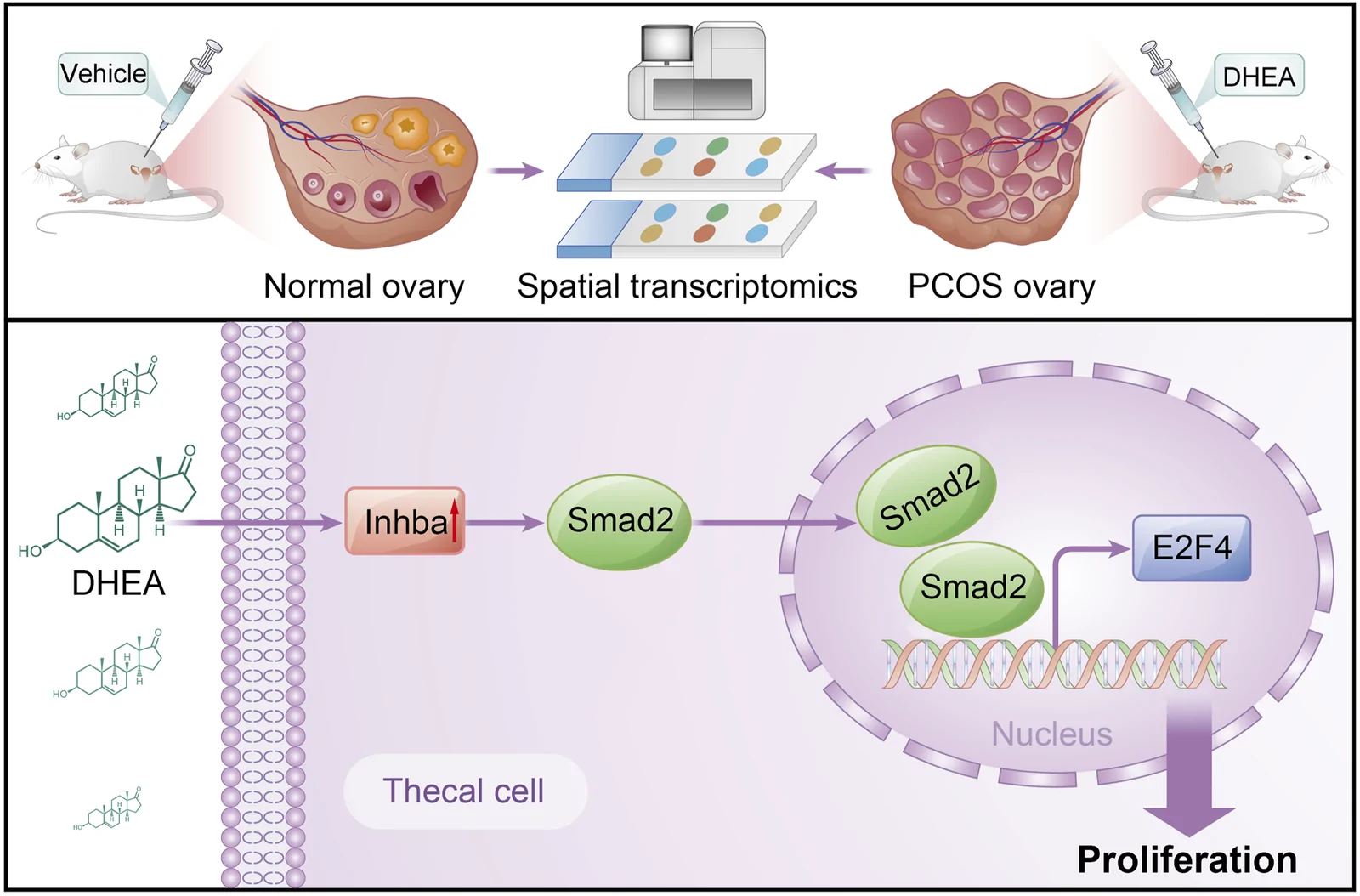
Schematic overview of the study design and proposed mechanism. Spatial transcriptomic profiling of ovaries from DHEA-induced PCOS mice revealed expansion of Lrp2^high^TC with elevated proliferative and steroidogenic activity. Mechanistically, DHEA stimulates Inhba expression, activating Smad2 and E2f4 signaling to drive thecal cell proliferation, contributing to androgen excess and ovarian dysfunction in PCOS.

To further support these findings, qRT-PCR and Western blot analyses were performed to evaluate gene expression levels (Figures 6C–I). DHEA significantly elevated the mRNA and protein expression of Inhba, Smad2, and E2f4, while gene-specific siRNA knockdown effectively suppressed both transcript and protein levels. Together, these results establish the Inhba/Smad2/E2f4 axis as a critical mediator of androgen-induced TCs proliferation and cell cycle progression in the PCOS model.

## Discussion

Excessive proliferation of TCs is a common feature of ovarian follicles in PCOS, closely linked to disrupted folliculogenesis under hyperandrogenism (Goodarzi et al., 2011; Azziz et al., 2016). As TCs are a major source of ovarian androgen biosynthesis, their hyperproliferation is fundamental to understanding the mechanisms underlying hyperandrogenism in PCOS. Although previous studies have employed bulk transcriptomics and single-cell RNA sequencing to investigate follicular dysfunction in PCOS, these approaches lack spatial resolution and fail to capture the spatial organization and interaction between different ovarian compartments. Spatial transcriptomics provides an opportunity to map gene expression within its histological context, offering insights into the spatial dynamics of molecular processes in PCOS, which remain insufficiently explored. In this study, we utilized ST to examine the mechanisms driving TCs proliferation in a DHEA-induced mouse model of PCOS. Our analysis identified the Inhba/Smad2/E2f4 axis as a central driver of TCs hyperproliferation, particularly within the Lrp2^high^ TC, emphasizing its involvement in this pathological feature of PCOS.

Our results demonstrate that Lrp2 expression is directly responsive to androgen stimulation, underscoring its potential role in the molecular mechanisms driving TCs hyperproliferation in PCOS. While Lrp2 has previously been associated with lipid metabolism and cellular signaling pathways (Liu et al., 2023; Schröder et al., 2023), its regulation by androgens within the ovarian context has not been previously established. In the current study, DHEA treatment significantly upregulated Lrp2 expression, particularly within the hyperproliferative Lrp2^high^TC subset, suggesting a specific link between androgen signaling and this cellular expansion. Prior research indicates that Lrp2 can mediate the uptake of steroid hormones, including testosterone and estradiol, through interactions with sex hormone-binding globulin (SHBG) (Balogh et al., 2019; Marzolo and Farfán, 2011). This suggests a potential interface between Lrp2 and systemic androgen dynamics. However, direct androgen-induced modulation of Lrp2 expression in ovarian tissue has not been reported. Further research is warranted to delineate the molecular interplay between androgen signaling and Lrp2 function, which may offer therapeutic insights for mitigating TCs proliferation and restoring ovarian homeostasis in PCOS.

In Lrp2^high^ TC, Cyp17a1 and Hsd3b1 are predominantly expressed, with Cyp17a1 showing particularly high levels, emphasizing this subpopulation’s role in androgen excess in PCOS. These genes, along with Cyp11a1, encode key enzymes (CYP11A1, CYP17A1, and HSD3B1) essential for androgen biosynthesis. CYP11A1 converts cholesterol to pregnenolone, CYP17A1 processes pregnenolone and progesterone into androgen precursors like DHEA, and HSD3B1 converts DHEA to androstenedione (Naamneh et al., 2022). The marked upregulation of Cyp17a1 highlights the role of androgen in feedback regulation of CYP17A1, a rate-limiting enzyme in androgen biosynthesis. Spatial transcriptomic data further localize Lrp2^high^ TC to regions of active androgen synthesis, underscoring their central role and potential as a therapeutic target to mitigate androgen excess in PCOS.

Moreover, our study identifies the Inhba/Smad2/E2f4 signaling axis as a pivotal regulatory pathway driving the proliferation of Lrp2^high^ TC, providing insights into the molecular underpinnings of PCOS. Inhba, a key component of ovarian function and the TGF-β signaling pathway, along with its downstream effector Smad2 and the cell cycle regulator E2f4, act in concert to promote aberrant TCs expansion. Spatial transcriptomics revealed enhanced co-localization of these molecules within Lrp2^high^ TCs in PCOS ovaries, underscoring their role in abnormal cellular proliferation and supporting prior evidence linking dysregulated TGF-β signaling to PCOS pathophysiology (Wang and Li, 2023; Wang S. et al., 2022) Functional studies further demonstrated that knockdown of Inhba, Smad2 or E2f4 significantly reduced DHEA-induced TCs proliferation, with the most pronounced effect observed upon Inhba silencing. This finding establishes Inhba as a critical upstream regulator within this proliferative axis and highlights its therapeutic potential. These findings advance existing knowledge by providing a spatially resolved transcriptomic perspective on the PCOS ovaries, enabling the identification of specific cellular subpopulations and their distinct gene expression profiles. The therapeutic implications of targeting the Inhba/Smad2/E2f4 axis are particularly interesting given the limitations of current PCOS treatments, such as insulin sensitizers and anti-androgenic agents (Armani et al., 2022; Dumesic et al., 2015; Escobar-Morreale, 2018), which fail to address the molecular mechanisms underlying TCs hyperplasia. Inhibiting this pathway could attenuate TCs over proliferation, reduce androgen production, and alleviate hyperandrogenism, a core symptom of PCOS. Notably, Inhba inhibitors emerge as promising candidates for reducing TCs hyperplasia, while Smad2 and E2f4 inhibitors could also be explored for their potential to modulate proliferative activity without impairing normal ovarian function. Collectively, our findings illuminate the Inhba/Smad2/E2f4 axis as a central driver of TCs proliferation in PCOS and underscore its potential as a target for developing molecular therapies aimed at addressing the underlying pathophysiology of this complex disorder.

While previous work from our group applied integrated single-cell and ST to characterize androgen-induced changes in the ovarian microenvironment, with a focus on granulosa and TCs remodeling through inflammatory and metabolic signaling pathways, the present study extends this line of investigation by emphasizing proliferative regulation within TCs. We identify a distinct Lrp2^high^ TC subpopulation that is markedly expanded in PCOS ovaries and demonstrate that its proliferation is regulated by a spatially co-localized Inhba/Smad2/E2f4 signaling axis. This pathway was further validated through gene knockdown experiments, providing mechanistic insight into the drivers of TCs hyperplasia.

These findings contribute to a broader understanding of androgen-associated ovarian alterations in a DHEA-induced PCOS model, integrating molecular, spatial, and functional dimensions. While the study was based on a well-established animal model, further validation in human tissues and across PCOS phenotypes is needed to assess its translational relevance. As the current work focused on local proliferative signaling, broader functional outcomes including folliculogenesis, ovulation, and systemic metabolic or inflammatory profiles were not addressed. Additionally, spatial validation of protein co-expression in ovarian tissue and interactions with other pathways such as MAPK or PI3K-AKT remain to be explored. Cluster abundance was assessed through UMAP and proportion analysis, approaches commonly applied in spatial transcriptomic studies. Future studies may incorporate statistical tools such as Milo, DAseq, or scCODA to enhance differential abundance analysis. Lrp2 was identified as an androgen-responsive marker of proliferative TCs. Although functional perturbation was not performed, its potential role in androgen signaling and TCs proliferation warrants further investigation. Overall, this study outlines a regulatory framework for TCs hyperplasia and provides a basis for future research aimed at addressing ovarian dysfunction in PCOS.

## Conclusion

In conclusion, our study highlights the role of Inhba/Smad2/E2f4 signaling axis in TCs proliferation in response to androgen (Figure 7). The expansion of Lrp2^high^ TC subset together with their upregulated cell cycle-related genes suggest that this axis plays a central role in the TCs hyperplasia. Targeting the Inhba/Smad2/E2f4 axis may control the TCs over proliferation as a potential therapeutic strategy for PCOS. Future research should aim to validate these findings in human tissues and explore their applicability across diverse PCOS phenotypes.

## Data Availability

The datasets presented in this study can be found in online repositories. The names of the repository/repositories and accession number(s) can be found below: https://www.ncbi.nlm.nih.gov/, GSE296728.

## Supplementary material

SUPPLEMENTARY FIGURE S1 Knockdown efficiency of Inhba, Smad2, and E2f4. **(A–C)** qRT-PCR analysis showing reduced mRNA levels after siRNA transfection. **(D–F)** Western blot confirming protein knockdown. GAPDH served as loading control. Data are represented as mean ± SD, *P < 0.05 by t-test.
