# Correction: Spatial transcriptomics reveals Inhba/Smad2/E2f4 axis in Lrp2^high^ thecal cell proliferation in androgen-induced PCOS mice

**DOI:** 10.3389/fcell.2026.1953043

**Published:** 2026-08-11

**Authors:** Man Luo, Xiaona Tian, Li Li, Guomei Zhang, Wenzhi Liu, Linlin Mei, Haoran Li, Xiaoyan You, Dongmei Zhang, Mengsi Zhou, Cheng Xiao, Jianping Ye, Xiaofeng Yang

**Affiliations:** 1 Department of Obstetrics and Gynecology, Zhengzhou Central Hospital Affiliated to Zhengzhou University, Zhengzhou, China; 2 Zhengzhou Key Laboratory of Endocrine Metabolism and Immunity in Polycystic Ovary Syndrome, Zhengzhou, China; 3 Institute of Muscle Biology and Growth, Research Institute for Farm Animal Biology (FBN), Dummerstorf, Germany; 4 Institute of Agricultural and Environmental Sciences, Rostock University, Rostock, Germany; 5 Institute of Trauma and Metabolism, Zhengzhou Central Hospital Affiliated to Zhengzhou University, Zhengzhou, China; 6 Tianjian Laboratory of Advanced Biomedical Sciences, Academy of Medical Sciences, Zhengzhou University, Zhengzhou, China

**Keywords:** polycystic ovwertttary syndrome, spatial transcriptomic, thecal cell, androgen, proliferation

An incorrect Funding statement was provided. The correct funding statement reads:

The author(s) declare that financial support was received for the research and/or publication of this article. This research was sponsored by Natural Science Foundation of Henan (252300421643), Zhengzhou Central Hospital Affiliated to Zhengzhou University (SR-0131), Zhengzhou Municipal Health Commission (ZZYK2024040), and Health Commission of Henan Province (LHGJ20240966).

The original version of this article has been updated.

