# Supplementary material for: Spatial transcriptomics reveals Inhba/Smad2/E2f4 axis in Lrp2high thecal cell proliferation in androgen-induced PCOS mice
Source: Front Cell Dev Biol. 2025 Aug 4;13:1633254. doi: 10.3389/fcell.2025.1633254 (PMC12358492; doi:10.3389/fcell.2025.1633254)
Supplement: Supplementary file 1 [file Table1.docx]

**Supplementary Table 1.** Spot Counts Before and After Quality Control Filtering

| **Slide** | **before_filter** | **after_filter** |
| --- | --- | --- |
| Slide1 | 1401 | 1400 |
| Slide2 | 1426 | 1426 |
| Slide3 | 776 | 773 |

**Note:** Slide1 includes Control samples 1-3; Slide2 corresponds to PCOS sample 1; Slide3 includes PCOS samples 2 and 3. Spot counts represent tissue-covered regions before and after quality control filtering (mitochondrial gene content >10% or detected genes <100).
