# Supplementary figures and images for: Spatial transcriptomics reveals Inhba/Smad2/E2f4 axis in Lrp2high thecal cell proliferation in androgen-induced PCOS mice
Source: Front Cell Dev Biol. 2025 Aug 4;13:1633254. doi: 10.3389/fcell.2025.1633254 (PMC12358492; doi:10.3389/fcell.2025.1633254)

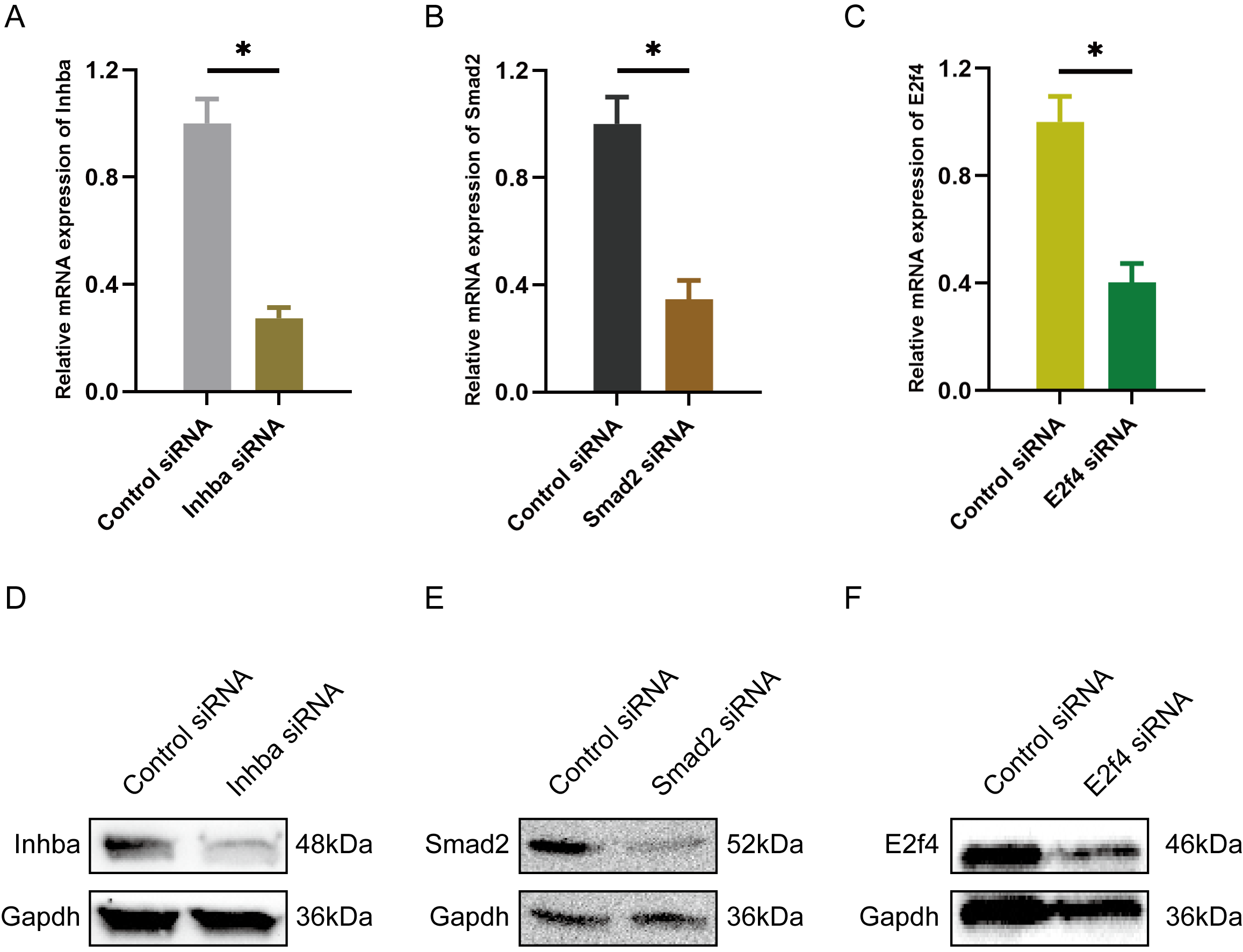

Supplement: Supplementary file 2 [file Image1.tif]
