# Supplementary material for: Spatial transcriptomics reveals Inhba/Smad2/E2f4 axis in Lrp2high thecal cell proliferation in androgen-induced PCOS mice
Source: Front Cell Dev Biol. 2025 Aug 4;13:1633254. doi: 10.3389/fcell.2025.1633254 (PMC12358492; doi:10.3389/fcell.2025.1633254)
Supplement: Supplementary file 3 [file Table2.docx]

**Supplementary Table 2.** Summary of spatial transcriptomic quality control metrics

|  | **Slide1** | **Slide2** | **Slide3** |
| --- | --- | --- | --- |
| **total spots** | 1401 | 1426 | 776 |
| **mean reads/spot** | 182587 | 244545 | 343215 |
| **Sequencing Saturation** | 78% | 77.38% | 80.86% |

**Note:** Slide1 includes Control samples 1-3; Slide2 corresponds to PCOS sample 1; Slide3 includes PCOS samples 2 and 3.
