# Supplementary material for: Spatial transcriptomics reveals Inhba/Smad2/E2f4 axis in Lrp2high thecal cell proliferation in androgen-induced PCOS mice
Source: Front Cell Dev Biol. 2025 Aug 4;13:1633254. doi: 10.3389/fcell.2025.1633254 (PMC12358492; doi:10.3389/fcell.2025.1633254)
Supplement: Supplementary file 4 [file Table3.docx]

**Supplementary Table 3.** Ct Values of Gapdh Across Experimental Groups

| **Group** | **Sample 1** | **Sample 2** | **Sample 3** | **Mean ± SD** | **CV (%)** |
| --- | --- | --- | --- | --- | --- |
| Control | 18.92 | 18.97 | 18.98 | 18.96 ± 0.03 | 0.16 |
| PCOS | 19.10 | 19.06 | 19.08 | 19.08 ± 0.02 | 0.10 |
| DHEA | 19.08 | 19.07 | 19.09 | 19.08 ± 0.01 | 0.05 |
| DHEA+*Inhba* siRNA | 19.01 | 19.03 | 19.02 | 19.02 ± 0.01 | 0.05 |
| DHEA+*Smad2* siRNA | 19.06 | 19.07 | 19.08 | 19.07 ± 0.01 | 0.05 |
| DHEA+*E2f4* siRNA | 19.10 | 19.11 | 19.09 | 19.10 ± 0.01 | 0.05 |

**Note:** Each Ct value represents one biological replicate (n = 3).
